# Histone lysine methyltransferase-related neurodevelopmental disorders: current knowledge and saRNA future therapies

**DOI:** 10.3389/fcell.2023.1090046

**Published:** 2023-02-27

**Authors:** Charlotte Roth, Helena Kilpinen, Manju A. Kurian, Serena Barral

**Affiliations:** ^1^ Molecular Neurosciences, Developmental Neurosciences Programme, Zayed Centre for Research into Rare Disease in Children, Great Ormond Street Institute of Child Health, University College London, London, United Kingdom; ^2^ Helsinki Institute of Life Science, University of Helsinki, Helsinki, Finland; ^3^ Faculty of Biological and Environmental Sciences, University of Helsinki, Helsinki, Finland; ^4^ Department of Neurology, Great Ormond Street Hospital for Children, London, United Kingdom

**Keywords:** neurodevelopmental disorders (NDDs), small-activating RNA (saRNA), histone lysine methyltransferases (HKMTs), epigenetics (chromatin remodeling), brain organoids

## Abstract

Neurodevelopmental disorders encompass a group of debilitating diseases presenting with motor and cognitive dysfunction, with variable age of onset and disease severity. Advances in genetic diagnostic tools have facilitated the identification of several monogenic chromatin remodeling diseases that cause Neurodevelopmental disorders. Chromatin remodelers play a key role in the neuro-epigenetic landscape and regulation of brain development; it is therefore not surprising that mutations, leading to loss of protein function, result in aberrant neurodevelopment. Heterozygous, usually *de novo* mutations in histone lysine methyltransferases have been described in patients leading to haploinsufficiency, dysregulated protein levels and impaired protein function. Studies in animal models and patient-derived cell lines, have highlighted the role of histone lysine methyltransferases in the regulation of cell self-renewal, cell fate specification and apoptosis. To date, in depth studies of histone lysine methyltransferases in oncology have provided strong evidence of histone lysine methyltransferase dysregulation as a determinant of cancer progression and drug resistance. As a result, histone lysine methyltransferases have become an important therapeutic target for the treatment of different cancer forms. Despite recent advances, we still lack knowledge about the role of histone lysine methyltransferases in neuronal development. This has hampered both the study and development of precision therapies for histone lysine methyltransferases-related Neurodevelopmental disorders. In this review, we will discuss the current knowledge of the role of histone lysine methyltransferases in neuronal development and disease progression. We will also discuss how RNA-based technologies using small-activating RNAs could potentially provide a novel therapeutic approach for the future treatment of histone lysine methyltransferase haploinsufficiency in these Neurodevelopmental disorders, and how they could be first tested in state-of-the-art patient-derived neuronal models.

## Introduction

Histone lysine methyltransferases (HKMTs) are part of the epigenetic system that regulates chromatin remodeling and ultimately gene expression. HKMTs are chromatin modifying enzymes that catalyze the transfer of a methyl group (CH_3_) from a donor molecule S-Adenosyl methionine (SAM) on the N-terminal of histone proteins (Murray, 1964; Cantoni, 1975; Finkelstein, 1990). This process, which was initially thought to be irreversible, has been now recognized to be part of a highly dynamic system involved in cellular renewal, specification of cell identity, cell differentiation, maintenance of DNA integrity, and DNA replication (Desrosiers and Tanguay, 1988; Benveniste et al., 1995; Aletta et al., 1998; Chen et al., 1999; Fuks et al., 2000; Sun et al., 2005; Pfister et al., 2014; Pai et al., 2017). Depending on tissue localization and biological context, histone methylation can result in transcriptional activation or repression (Strahl et al., 1999; Aymard et al., 2014; Salinas et al., 2020). Mutations in *HKMT* genes have been reported to cause childhood neurodevelopmental disorders (NDDs) (Kuroki et al., 1981; Jones et al., 2012; Koemans et al., 2017) and several form of cancers in childhood (Attarbaschi et al., 2022) and adulthood (Turkmen et al., 2012; Gala et al., 2018; Alam et al., 2020).

Studies in cancer have improved our understanding of the role of HKMTs in the regulation of cellular processes. It is hypothesized that epigenetic dysregulation could precede or initiate the neoplastic process (Fraga et al., 2005; Kuo et al., 2011). HKMTs are commonly overexpressed in cancers, leading to an imbalance of epigenetic regulation, either by inadequate activating or repressing gene expression (Barlesi et al., 2007; Bianco-Miotto et al., 2010). For some HKMTs, downregulation of chromatin remodelers has been related to poor prognosis, higher metastatic risks and drug resistance (Cheng et al., 2001; Ruault et al., 2002; Lee et al., 2009; Morin et al., 2011; Watanabe et al., 2011; Chen et al., 2014; Will and Steidl, 2014). Importantly, the role of HKMT in cancer has also been attributed to their capacity to methylate non-histone targets, as described for tumor suppressor p53 which requires HKMT-mediated methylation to be activated (Lee et al., 2009; Li et al., 2020). Not surprisingly, the role of HKMT in cancer has accelerated the design of novel therapies aiming to modulate their expression and restore physiological levels (Gilbert et al., 2004; Barlesi et al., 2007), for example, by using HKMT inhibitors (Cameron et al., 1999; Baylin and Jones, 2011).

Due to their key role in regulating early stages of human development (Xu et al., 2019), it is not surprising that germline mutation in HKMT genes are frequently reported in NDDs. It is now well recognized that *de novo* mutations in HKMT genes can lead to haploinsufficiency with reduced protein expression and impaired protein function (Faundes et al., 2018). Here, we will provide a brief overview of the role of HKMTs, both in normal cellular function and NDD pathophysiology. We will focus on the KMT2 family, for which several monogenic diseases have been described. We will discuss how advances in small-activating RNA (saRNA) technologies could potentially be exploited as a novel precision therapy approach for KMT2 haploinsufficiency. Finally, we will discuss how these therapeutic strategies could be further investigated in patient-derived neuronal models, enabling preclinical development of saRNA-based novel therapies for this group of diseases.

## HKMT haploinsufficiency in NDDs

NDDs encompass a broad heterogeneous spectrum of disabling neurological diseases characterized by impairment of one or more aspects of human development (Pichot, 1986). By definition, they present in childhood, usually during the early years of infancy or childhood (Howlin et al., 2004; Morris-Rosendahl and Crocq, 2020). Neurological features such as intellectual disability are a core component of NDDs; however, NDDs are often multisystemic disorders with a complex pathophysiological phenotype (Kim and Leventhal, 2015; Niemi et al., 2018). Recent epidemiological studies have highlighted the co-occurrence of multiple neurological phenotypes in 70% of people with NDDs, complicating their classification and diagnosis (Fombonne, 2003; Straub et al., 2022). Despite advances in diagnostic tools such as next-generation sequencing technologies, many NDDs still remain undiagnosed. Therefore, the overall disease incidence is estimated to be higher than currently reported (Deciphering Developmental Disorders Study, 2017; Frances et al., 2022; Straub et al., 2022). Collectively, the social, economic and personal costs of NDDs present significant burden to both families and society. For the great majority, there are no disease-modifying treatments and care consists solely of symptomatic and palliative management (Thapar et al., 2017; Ismail and Shapiro, 2019). Etiologically, NDDs may be broadly divided into those predominantly caused by environmental factors (including birth injury, nutritional defects, infections) and genetic factors. Epigenetic factors are also likely to play a role in some NDDs (Lister et al., 2013; Salinas et al., 2020). An increasing number of NDDs have recently been reported to be caused by *de novo* mutations in HKMT genes (Ciptasari and van Bokhoven, 2020; Reichard and Zimmer-Bensch, 2021). Different forms of NDDs have been associated with loss-of-function mutations leading to haploinsufficiency (Singh et al., 2016; Zech et al., 2016; Deciphering Developmental Disorders Study, 2017; Yu et al., 2019; Kummeling et al., 2021).

HKMTs are ubiquitously expressed, highly conserved enzymes responsible for the methylation of lysine residues positioned along histone tails emerging from the nucleosome. Nucleosomes are made of four pairs of histone proteins: H2.A, H2.B, H3 and H4 around which the DNA is wrapped, allowing close interaction between DNA and histone proteins (Richmond et al., 1984; Arents and Moudrianakis, 1993). Methylation of the lysine residues of histones is a post-translational modification (PTM) which acts in synergy with other PTMs such as acetylation, ubiquitylation and phosphorylation, allowing interaction with a range of cofactors involved in the regulation of gene transcription (Fuks et al., 2000; Strahl and Allis, 2000; Ballestar and Esteller, 2002; Wang et al., 2004; Chaumeil et al., 2006). Notably, PTMs such as acetylation and phosphorylation are responsible for introducing a positive charge to histone proteins and directly affect the position of the histone tails (Bradbury, 1992). Histone methylation shapes the conformation of the chromatin through recruitment and interaction with complexes regulating the state of chromatin compaction and thus accessibility to DNA (Chen et al., 1999; Greer and Shi, 2012). Overall, the chromatin state is crucial to spatially control the access of transcription machinery to specific genes responsible for cellular physiology and cell identity. Although there are exceptions, methylation of the lysine 4 and 36 of the histone protein 3 (H3K4 and H3K36, respectively) activate gene transcription (Strahl et al., 1999; Heintzman et al., 2007; Aymard et al., 2014), whereas methylation in histone three lysine residues 9, 27, 79 (H3K9, H3K27, H3K79) and in histone four lysine 20 (H4K20) are more likely to be found in transcriptionally inactive heterochromatin domains (Tachibana et al., 2001; Lachner and Jenuwein, 2002; Shi et al., 2003; Peinado et al., 2004; Yang et al., 2008). Adding to the intricacy of this regulatory system, lysine residues can be mono-, di- or tri-methylated (Murray, 1964; Paik and Kim, 1967; Hempel et al., 1968). Each HKMT-related PTM at specific amino acid residues has been associated with different functions (Table 1).

**TABLE 1:** Histone lysine methyltransferases causing neurodevelopmental disorders and their relevant cell/animal models.

HKMT gene	Targets	Neurodevelopmental phenotype (inheritance/penetrance/prevalence^a^)	Laboratory models
*KMT2A/MLL1*	H3K4me1/2/3	Wiedemann Steiner Syndrome (*de novo*/autosomal dominant/1/25,000 - 40,000) (Jones et al., 2012; Reynisdottir et al., 2022); (Sheppard and Quintero-Rivera, 1993)	Conditional deletions in mice: cognitive and behavioral alterations (spatial working memory, nest building, anxiety); reduction of H3K4me3 at promoters of genes associated with neuropsychiatric susceptibility in cortical neurons; reduced H3K4me1 at enhancers (Jakovcevski et al., 2015; Kerimoglu et al., 2017; Michurina et al., 2022)
			KO mouse embryonic fibroblasts: reduction of H3K4me2/3 and reduced expression of *HoxA9* (Gregory et al., 2007)
*KMT2B/MLL2/WBP7*	H3K4me1/2/3	KMT2B-related dystonia (DYT18) (*de novo* or inherited/autosomal dominant/) (Zech et al., 2016; Meyer et al., 2017) KMT2B non-dystonia neurodevelopmental phenotype (Cif et al., 2020)	Conditional KO in mouse excitatory forebrain neurons: hippocampus-dependent learning impairment (short and long-term memory), reduction of H3K4me1 (Kerimoglu et al., 2013; Kerimoglu et al., 2017; Michurina et al., 2022)
			Patient-derived fibroblasts: endo-lysosomal processing abnormalities (cholesterol and sphingolipid accumulation) (Zhao et al., 2018)
*KMT2C/MLL3/HALR*	H3K4me1 (Cho et al., 2007; Hu et al., 2013)	Kleefstra Syndrome 2 (*de novo*/autosomal dominant) (Koemans et al., 2017; Siano et al., 2022)	Double KO of mouse embryonic stem cell, kmt2c KO and conditional kmt2d KO: reduction of H3K4me1/2 (Wang et al., 2016)
*KMT2D/MLL4 (MLL2)/ALR*	H3K4me1 (Hu et al., 2013)/2/3	Kabuki Syndrome (*de novo* or inherited//autosomal dominant/1/32,000) (Kuroki et al., 1981; Ng et al., 2010); (Adam et al., 1993)	Conditional KO mice and immortalized BAT: diminution of muscle mass and BAT, dysregulation of genes involved in adipocytes and myocytes differentiation, reduction of H3K4me1/2 at enhancers (Lee et al., 2013)
			Double KO mouse embryonic fibroblasts, kmt2c KO and conditional kmt2d KO: reduction of H3K4me1/2 (Wang et al., 2016)
			KO in Human B-cell culture (Gm12878 cells) by siRNA: downregulation of the ITGB7 transcript. Kmt2d+/bGeo mouse: immunodeficiency (decrease of IgA and Peyer patches) (Pilarowski et al., 2020)
*KMT2F/SETD1A/SET1A*	H3K4me1/2/3 (Wang et al., 2021)	DD, ID, epilepsy, and Schizophrenia (*de novo o*r inherited/autosomal dominant) (Yu et al., 2019; Kummeling et al., 2021; Singh et al., 2022)	KO model of SCZ: cognitive impairment (working memory abnormality, synaptic malfunctions) (Mukai et al., 2019; Nagahama et al., 2020)
			Haploinsufficiency hiPSC-derived neuronal model using CRIPSR-cas9: impairment of neuronal structure (increase of dendritic length and arborization), function (increase burst activity, synaptic integration), and molecular mechanism (increase cAMP level) (Wang et al., 2022)
*KMT2G/SETD1B/SET1B*	H3K4me3	IDDSELD (*de novo*/autosomal dominant) (Labonne et al., 2016; Hiraide et al., 2019; Michurina et al., 2022)	Post-natal conditional deletions in murine forebrain excitatory neurons: hippocampus-dependent learning impairment, diminution of H3K4me3 near transcription start site (TSS) of genes implicated in neuronal plasticity (Michurina et al., 2022)
*KMT2H/ASH1L*	H3K4, (Gregory et al., 2007) H3K36me1/2 (Tanaka et al., 2007; Miyazaki et al., 2013)	ID, ASD, seizures (*de novo o*r inherited/autosomal dominant)/(De Rubeis et al., 2014; Stessman et al., 2017)	Neuronal KO using inducible RNAi in *Drosophila* model: ASD and ID behavioral assay with impairment of higher cognitive functions (Stessman et al., 2017)
			KD in HEK-293 T with shASH1L (Gregory et al., 2007): reduction of H3K4me3 with reduce expression of Hox genes
			KO using morpholino antisense oligonucleotides in zebrafish: reduction of epiphysial neurogenesis, and abnormality of cell fate with impairment of photoreceptors and projection neurons (Cau and Wilson, 2003)
			KO using the gene trap mouse line: reduced viability, size and fertility (Brinkmeier et al., 2015)

Abbreviations: ASD, autism spectrum disorder; ASH1L, Absent Small And Homeotic Disks Protein one Homolog Like KMT); BAT, brown preadipocytes; DD, developmental delay; HCC, hepatocellular carcinoma; HEK, embryonic kidney 293; hiPSC: human induced pluripotent stem cells; HKMTs, histones lysine methyltransferases; ID, intellectual disability; IDDSELD, intellectual developmental disorder with seizures and language delay; KD, knockdown; KMT, lysine methyltransferase; KO, knockout; MCA, multiple congenital anomalies; MLL, Mixed lineage leukemia protein-1; RNAi, RNA, interference; SCZ, schizophrenia; SETD1A/1B, SET, Domain Containing 1A/1B, histone lysine methyltransferase; shASH1L, short hairpin ASH1L; WHS, Wolf-Hirschhorn syndrome.

^a^The estimated prevalence (when known) is reported based on the orphanet database.

One of the most studied subgroups of HKMTs is the lysine methyltransferase 2 (KMT2) family, which methylates the lysine four of the histone 3 (H3K4) *via* its catalytic SET (Su(var) 3-9, Enhancer-of-zeste, Trithorax) domain (Rea et al., 2000). Mono-methylation of H3K4 (H3K4me) is mostly present in enhancers (Heintzman et al., 2009), di-methylation (H3K4me2) is found throughout active genes and tri-methylation (H3K4me3) is enriched at active promoters (Guenther et al., 2005; Heintzman et al., 2007). Crosstalk between the different methylation states, other histones PTMs and single proteins or protein complexes is essential for genomic transcription.

**FIGURE 1:**
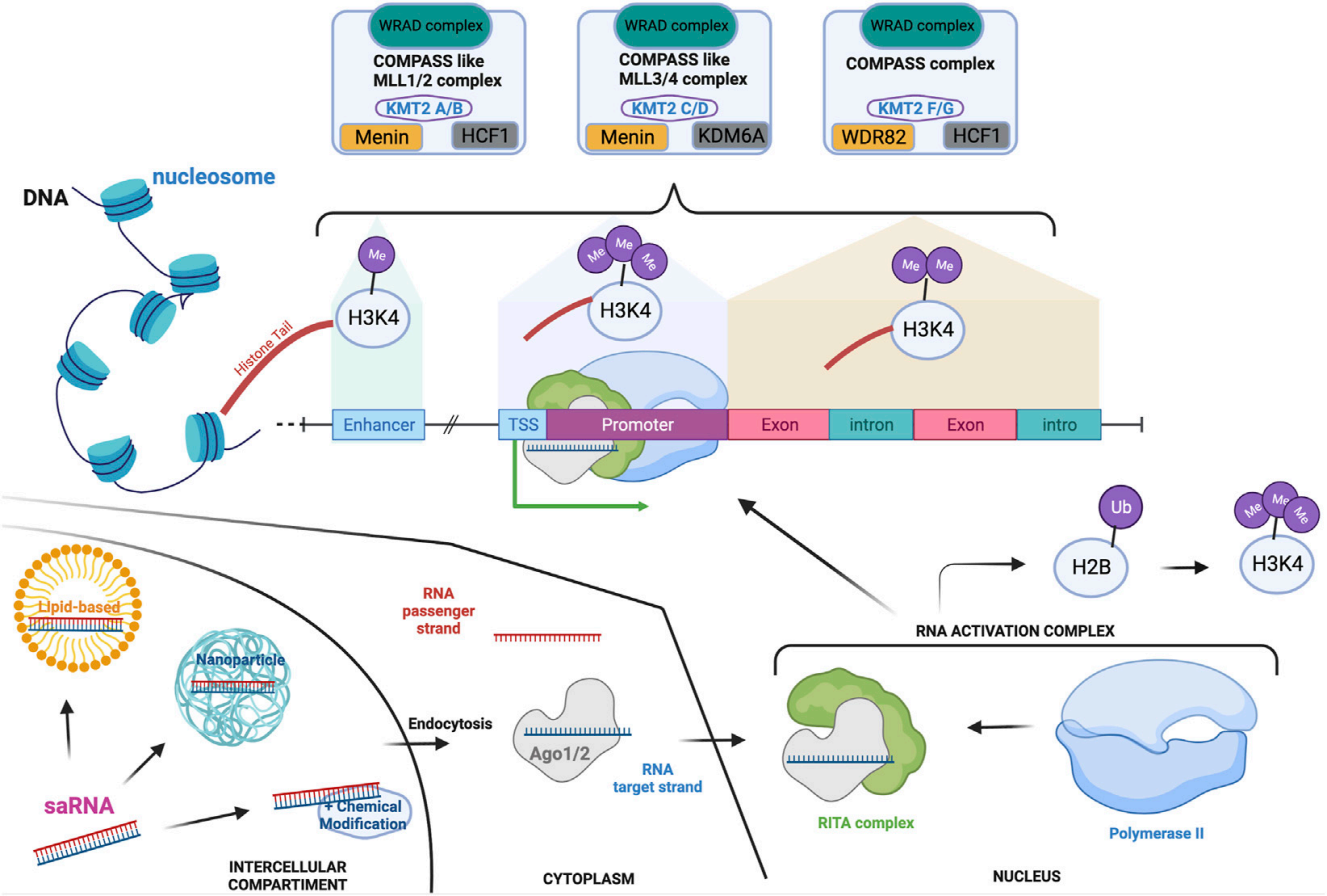
KMT2-associated complexes and saRNA mode of action in the transcription machinery. Transcription activation is a unique mechanism that is in part controlled by the addition of mono-(at enhancer), di- (at actively transcribe gene locus) or tri-(at promoter) methylation on the lysine four of the histone 3. These methylations are mediated by the COMPASS and COMPASS-like complexes which are formed by the association of specific subunits. Each of the complexes hold a common subunit called WRAD to which associate specific subunits: Menin and HCF1 to generate the COMPASS like/MLL1-2 complexes; Menin and KDM6A for the COMPASS like/MLL3-4 complex; HCF-1 and WDR82 for the COMPASS complex. saRNAs are synthetic RNA probes which enable transcription activation. Double-stranded saRNA can be delivered by various vehicles to the target cell, such as lipid-based or nanoparticle technology. After endocytosis, the single RNA target strand is loaded on the Ago protein before translocating to the nucleus. The loaded Ago protein associates with the RITA complex to recruit polymerase II and form the RNA activation complex that is guided to the promoter of the target gene. This is associated to ubiquitination of the histone 2 b which then triggers H3K4me3, enhancing the activation of transcription. If targeting HKMTs, this will increment their transcription and transduction enabling mono-, di- or tri-methylation of specific lysine (K) on histone (H) tails. Depending on the lysine localization, these post-translational modifications will repress or activate specific genes. Created with BioRender.com.

Six members of the KMT2 family interact within the Complex of Proteins Associated with Set1 (COMPASS) and COMPASS-like complexes (also called MLL complexes), composed of ‘writers’ (methyltransferase), ‘erasers’ (demethylase) and ‘readers’ (Park et al., 2019; Lavery et al., 2020). Importantly, the subunit ‘WRAD’, composed of WD repeat domains 5 (WDR5), retinoblastoma binding protein 5 (RrBP5), absent small or homeotic 2-like (ASH2L), and dumpy-30 (DPY-30), is a subunit present across each of the MLL complexes and which enhances the methylation capability as well as substrate specificity (Steward et al., 2006; Cho et al., 2007; Cao et al., 2010; Dharmarajan et al., 2012). Although KMT2A and B, KMT2C and D, KMT2G and H are respectively paralogues, they have been shown to have stringent substrate specificity due to their association with distinct co-factors (Figure 1) (Hughes et al., 2004; Cho et al., 2007). As a result, mutations in each of the *KMT2* genes have been linked to different NDDs, with distinct but overlapping features.

Mutations in *KMT2A* have been linked to Wiedemann Steiner Syndrome (WSS), an autosomal-dominant disorder that presents with intellectual disability (ID), facial dysmorphism, hypertrichosis, motor delay and hypotonia (Steiner and Marques, 2000; Jones et al., 2012). Most WSS patients have premature stop-codon mutations in the enzymatic SET domain, leading to loss-of-function and haploinsufficiency (Jones et al., 2012). However, more recently, some variants have been identified in the CXXC domain which result in increased binding at unmethylated CpG islands of DNA, leading to *KTM2A* overexpression and abnormal nuclear localization (Lebrun et al., 2018). Artificial intelligence (AI) systems are now facilitating the analysis of variant pathogenicity; AlphaFold2 can predict the effect of different variants of the KMT1A-CXXC domain on the 3D conformation of methyltransferases (Tunyasuvunakool et al., 2021; Reynisdottir et al., 2022).

Haploinsufficiency of *KMT2B* causes KMT2B-related dystonia as well as a neurodevelopmental (non-dystonic) phenotype (Zech et al., 2016; Meyer et al., 2017; Faundes et al., 2018). Since its discovery as a disease-causing gene, over a hundred pathogenic variants have been identified, making it one of the most common genetic etiologies of complex childhood onset dystonia (Gorman et al., 2018; Cif et al., 2020; Damasio et al., 2021; Grosz et al., 2022). KMT2B-related dystonia usually presents early in childhood, with lower limb dystonia progressing into a generalized dystonia with prominent cervical, cranial, and laryngeal involvement (Zech et al., 2016; Meyer et al., 2017). DNA methylation profiling reveals a unique methylation signature in KMT2B-related disease, which not only aids in determining the pathogenicity of *KMT2B* variants of uncertain significance (VUS) but could also be predictive of the onset and severity of disease (Ciolfi et al., 2021). Such methylation signatures also differentiate KMT2B-related disease from Kabuki syndrome, which has a different methylation (Lee et al., 2022).

Although KMT2A and KMT2B are phylogenetically closely related, and both are responsible for the methylation of the H3K4 residue, systematic studies have highlighted unique roles for each (Kerimoglu et al., 2017). For instance, a recent study of knock-out (KO) mouse models of these two methyl transferases showed that both *KMT2A* and *KMT2B* are associated with memory consolidation (Kerimoglu et al., 2013; Kerimoglu et al., 2017). However, RNA sequencing and Chromatin Immunoprecipitation Sequencing (ChIP-seq) analysis have shown limited overlap of the gene expression profiles between *KMT2A* and *KMT2B-*KO mice (Michurina et al., 2022). Moreover, these studies have highlighted that the observed reduction of H3K4me3 at specific promoters was dependent on the specific KMT2-KO (Kerimoglu et al., 2013; Kerimoglu et al., 2017). KMT2B, and not KMT2A, seems to have a pivotal role at bivalent promoters (Tomizawa et al., 2018). Bivalency is a chromatin state where repressive (H3K27) and active (H3K4) marks co-occur at specific promoters, which are hypothesized to control bivalent genes that arekey regulators of either retaining stem cell identity or initiating cellular differentiation processes (Macrae et al., 2022).

Both KMT2C and KMT2D are histone methyltransferases of H3K4 and primarily target gene enhancers (Hu et al., 2013). Monoallelic *KMT2C* variants have recently been described in Kleefstra syndrome type 2 (Cheema et al., 2022; Siano et al., 2022). Notably, Kleefstra syndrome type 1 is caused by deletions in the sub-telomeric region of chromosome 9q which contains the *KMT1D* gene, a HKMT protein that mono- and di-methylates H3K9 (Kleefstra et al., 2006; Kleefstra et al., 2009). Patients with Kleefstra syndrome present with NDD, microcephaly, ID, autism spectrum disorder (ASD), and characteristic facial features, which are associated with hypotonia and heart defects in half of the cases, and epilepsy in a fifth of the patients. In contrast, mutations in *KMT2D* lead to a NDD called Kabuki Syndrome (KS) type 1 (Kuroki et al., 1981), a multisystemic disorder associated with developmental delay, short stature, variable ID, increased susceptibility to infection, risk of autoimmune disorders and characteristic facial dysmorphism (Stagi et al., 2016; Adam et al., 2019; Pilarowski et al., 2020). Most of the reported cases of KS are due to loss-of-function monoallelic mutations in *KMT2D*, but surprisingly, the second most frequent mutated gene is the X-linked Lysine-Demethylase-6A (*KDM6A*), an enzyme with quite different functions to KMT2D (Lederer et al., 2012; Micale et al., 2014). Both KMT2D and KDM6A are subunits of the MLL4/COMPASS-like complex (Figure Figure1), which could explain why they lead to phenotypically similar syndromes in the disease state.

Monoallelic variants in *KMT2F*, have been linked to schizophrenia (SCZ) as well as NDD, ID, and early onset epilepsy (Singh et al., 2016; Yu et al., 2019; Kummeling et al., 2021). Kummeling et al. described a neurodevelopmental syndrome caused by heterozygous loss-of-function mutations in *KMT2F*, presenting with ID, NDD, visual and/or hearing defects and psychiatric symptoms (Kummeling et al., 2021). Recently, using a humanised induced pluripotent stem cell (iPSC)-derived neuronal model of SCZ, Wang and others have elegantly demonstrated that haploinsufficiency of *KMT2F* leads to major neuronal network reorganization and modification of neuronal morphology (Table 1) (Wang et al., 2022).

*KMT2G* dysfunction has been linked to autosomal dominant NDD that typically presents with DD, ID, ASD, and drug-resistant early onset epilepsy (Labonne et al., 2016; Hiraide et al., 2019; Roston et al., 2021). Dysregulation of methylation has been shown in patients with characteristic hypermethylation signatures (Krzyzewska et al., 2019). Recently, the postnatal effect of loss-of-function of KMT2G have been explored in a conditional KO mice model and highlighted defects in H3K4me3 deposition in genes involved in neuronal plasticity, which affected hippocampus-dependent learning (Michurina et al., 2022).

KMT2H differs from the rest of the KMT2 family with regard to substrate. It has been reported that KMT2H methylates H3K4 (Gregory et al., 2007) as well as H3K36 (Tanaka et al., 2007; An et al., 2011; Miyazaki et al., 2013; Okamoto et al., 2017). The crystal structure suggests the existence of an auto-inhibitory loop (An et al., 2011), which is potentially inhibited upon interaction with the nucleosome. Monoallelic variants have been identified in patients with ID, ASD and seizures.

## SaRNA-based therapies as a potential precision treatment for NDDs

To date, treatment of NDDs related to defective HKMTs has been limited to symptomatic management (Thapar et al., 2017; Ismail and Shapiro, 2019). NDDs caused by monoallelic loss-of-function mutations represent an attractive group of disorders that could benefit from a strategy aiming to restore baseline gene expression by increasing the transcription of the healthy non-mutated allele.

SaRNAs are double stranded, small non-coding activating RNAs made of 21 nucleotides with a two-base overhang on each end (Li et al., 2006). Despite being structurally identical to small-inhibiting RNA (siRNA), they have the opposite effect of increasing target gene expression by a mechanism called RNA activation (RNAa) (Huang et al., 2010; Voutila et al., 2017). RNAa is mediated by an oligonucleotide sequence complementary to the promoter of the gene of interest. Binding of the saRNA to its complementary promoter sequence mediates target gene upregulation (Janowski et al., 2007). RNAa was first reported in 2006 using synthetically designed saRNAs, which were able to increase the transcription of a specific gene with high specificity and efficacy (Li et al., 2006). After entering a cell *via* endocytosis, one strand (either the sense or antisense, depending on cell type) of the saRNA is loaded to a protein of the Argonaut family (Ago1 or Ago2) and guided to its targeted promoter in the nucleus (Janowski et al., 2006; Portnoy et al., 2016) (Figure Figure1). This is another major difference with siRNA, which are loaded on Ago1-4 (Schwartz et al., 2008; Fimiani et al., 2016). Once in the nucleus, the Ago carrying the saRNA strand associates with the RNA-induced transcriptional activation (RITA) complex which recruits RNA polymerase II and promotes transcription (Portnoy et al., 2016). Additionally, it has been proposed that PTMs -such as ubiquitination of H2B that promotes methylation of H3K4 - participate in saRNA/Ago2-mediated transcriptional activation (Portnoy et al., 2016). Interestingly, although Ago1 has been primarily implicated in gene silencing, in some cases, Ago1 is key for the function of saRNAs. Indeed, the use of an Ago1 inhibitor leads to a complete loss of saRNA activity (Fimiani et al., 2016). Notably, mutations in *Ago2* and *Ago1* have also recently been associated with NDDs (Lessel et al., 2020; Schalk et al., 2022).

While several RNA-based strategies [such as antisense oligonucleotide (ASO), micro-RNA (miRNA), small interfering RNA (siRNA) short hairpin RNAs (shRNAs)] aim to *inhibit* expression of disease-causing genes (Matzke and Birchler, 2005; Lanford et al., 2010; Shen et al., 2012), Li and others’ study first allowed the exploration of RNA-based therapeutic strategies to increase expression of the target gene (Li et al., 2006). Moreover, their strategy showed that the saRNA-mediated transcriptional activation increased the protein production only in cells where the target gene was in a non-condensed accessible state, with limited if not null binding to genes in the euchromatin state. Therefore, saRNA are mostly functional in cells that physiologically express the target gene. This characteristic could potentially avoid off-target effects by only targeting cells in a specific cellular state where the gene of interest is expressed.

Soon after saRNA discovery, increased expression of the progesterone receptor (PR) was successfully achieved in two breast cancer cell lines (MCF7 and T47D) using a saRNA construct targeting the PR promoter (Janowski et al., 2007). The saRNA increased PR expression up to 18-fold compared to untreated cells. The addition of a deacetylase inhibitor and a methyltransferase inhibitor reduced the saRNA effect, indicating the potentially important role of PTMs in fine tuning gene expression *via* RNA activation. The efficacy of the saRNA construct was significantly higher when the sequence was designed to have 100% complementary with the target sequence, while saRNA sequences containing mismatched or scrambled saRNAs showed less or no efficacy, respectively (Janowski et al., 2007). The specificity of the saRNA resides in its seed region, which span from the second to the eighth nucleotide commencing at the 5′end, where a single nucleotide mismatched within this region could lead to decreased or null activity. Variants located outside of the seed region are more tolerated but may hamper saRNA functionality (Meng et al., 2016; Voutila et al., 2017).

In a very recent study, Andrikakou and others tested saRNA technology to increase the expression of a histone deacetylase, Sirtuin 1 (encoded by *SIRT1*) (Andrikakou et al., 2022). *SIRT1* is known for its protective effect on age-related disorders such as metabolic syndrome (Pfluger et al., 2008). Using a high fat diet rat model, they injected a saRNA against *SIRT1* conjugated with an aptamer to enable systemic delivery and showed a reduction of 0.6-fold decrease of serum cholesterol, 0.7-fold decrease of triglycerides and an increase of 1.6-fold increase of HDL/LDL ratio. They also observed a significant decrease in intracellular lipid accumulation and a reduction of rat weight (Andrikakou et al., 2022). This study therefore demonstrated well-tolerated systemic delivery of saRNA *in vivo,* without noticeable toxicity.

SaRNAs have also been tested *in vivo* in a mouse model of a Rett-like condition to correct haploinsufficiency of *Foxg1* (Fimiani et al., 2016). *FOXG1* is a transcription factor implicated in cortical development with a role in the maintenance of neural cells in the precursor state (Hanashima et al., 2004; Martynoga et al., 2005). Lentiviral delivery of eight sense and antisense saRNAs to a primary culture of E12.5 murine neocortical precursors showed an increase of *Foxg1* expression with all constructs tested (Fimiani et al., 2016). Moreover, saRNAs were able to induce the same results in mouse embryonic fibroblast (NIH3T3) and human embryonic kidney 293 (HEK) cell lines (Fimiani et al., 2016). To further analyze the potency of the saRNA on differentiating cells and their effect on the differentiation process, an inducible construct was delivered to E16.5 murine neocortical precursors. Conditional activation in cultured neocortical precursors over 5 days induced *Foxg1* expression and a significant reduction in Tubulin beta class III (TUBB3) in postmitotic neurons (Fimiani et al., 2016). Thus, this saRNA strategy was able to decrease neuronal maturation downstream of increased *Foxg1* expression. Interestingly, only one of the selected saRNAs was effective in E16.5 mouse primary neuronal cultures. This highlights the limited efficacy of saRNAs on cells where the baseline expression of the target gene is constitutively low. Intraventricular injection into neonate mouse pups with AAV-saRNA lead to a 1.66-fold increase in the mRNA expression of *Foxg1*, despite low infection efficiency (Fimiani et al., 2016).

Improvements in saRNA technology have led to the first clinical trial for the treatment of hepatocellular carcinoma using saRNA and nanoparticle-mediated delivery-NOV340-SMARTICLES (NCT02716012) (Sarker et al., 2020; Hashimoto et al., 2021). The therapeutic strategy is based on the MTL-CEBPA (MiNA therapeutics) - an saRNA targeting the CCAAT/enhancer-binding protein alpha (CEBPA) that codes the transcription factor C/EBP (Voutila et al., 2017; Reebye et al., 2018). MTL-CEBPA was first tested on a cirrhotic rat model (Reebye et al., 2014), then on human hepatocellular carcinoma cells HepG2 (Voutila et al., 2017) and finally in a rodent model (Reebye et al., 2018). MTL-CEBPA successfully increases CEBPA protein expression, leading to an increase in albumin and a reduction in tumor mass (Reebye et al., 2018). Interestingly, another group demonstrated the anti-inflammatory effect of MTL-CEBPA in an *E. coli*-derived lipopolysaccharide-challenged humanized mouse model (Zhou et al., 2019), demonstrating the potential versatility of saRNA.

Despite improvements and efforts to further optimize this technology, clinical translation of saRNAs still present some limitations. Indeed, as an RNA molecule, saRNAs are highly susceptible to degradation, which impacts the duration of therapeutic effect–in reality this will likely require repeated regular drug delivery to patients. Delivery of the saRNA remains one of the biggest challenges for efficient therapeutic translation of this technology. When systemic delivery is used, the saRNA are subjected to several processes, which drives their degradation, including renal clearance, nucleases, and lysosomal degradation (Alexis et al., 2008; Petros and DeSimone, 2010). Significant efforts have been made to reduce degradation of ASOs and siRNAs, and these will hopefully also translate to saRNA technology.

Another important aspect to consider is brain delivery of saRNA-based strategies for the treatment of NDDs. To date, delivery has been achieved for chemically modified RNA-based therapies *via* intrathecal (IT) or intracerebroventricular (ICV) injections (Hache et al., 2016; Hirunagi et al., 2021). This same administration route could potentially be used for saRNAs, although given that the effect of saRNAs is time-limited, to achieve a long-lasting effect, repetitive administration would be needed. Whilst ventricular reservoirs are also potentially another delivery route, ideally a systemic saRNA that crosses the brain blood barrier (BBB) to reach the specific brain areas would be even more desirable.

Current strategies to improve the half-life of RNA-based technologies rely on chemical modifications of the nucleobase, the backbone or ribose of the oligonucleotide (Roberts et al., 2020). Addition of phosphorothioate (PS) linkages affects the backbone, stereochemistry, and resistance to nucleases (Brown et al., 1994). However, these modifications, mainly tested in siRNA, will decrease affinity with the target gene, causing non-specific binding and global non-antisense effect, thus affecting overall efficiency and more importantly, safety (Brown et al., 1994; Guvakova et al., 1995; Giles et al., 1998; Liang et al., 2014; Liang et al., 2015). Regarding saRNAs, the 2ʹ-*O*-methyl or 2ʹ-Fluoro chemical modifications showed better results (Place et al., 2010; Kang et al., 2012); 2ʹ-Fluoro modified P21 targeting saRNA, when compared to its non-modified counterpart, showed an increase resistance to nuclease and reduced immunogenicity whilst maintaining efficacy. The non-immunogenicity of 2ʹ-Fluoro modification represents one of the major aspects for clinical translation of this technology. In addition, the use of lipid nanoparticles for intravesical delivery in mice bladder did not modify the saRNA measured activity (Kang et al., 2012). Indeed, other major technological advances have allowed saRNA molecules to associate with peptides, lipids, antibodies and nanocarrier liposomes to improve their stability (Kang et al., 2012; Blakney et al., 2019; Zhou et al., 2019).

When developing saRNA-based therapies for NDDs, some important aspects should be considered for preclinical proof-of-concept studies. HKMT-related NDDs present with complex developmental phenotypes. Animal models do not always recapitulate features of human disease, likely due to key differences between human and murine brain development. It would therefore be potentially advantageous to validate saRNAs in systems that more closely mimic the human brain. Patient-derived neuronal models represent an ideal platform for the development of saRNA therapies for NDDs. Since the first generation of two-dimensional (2D) iPSC-derived neuronal models, technological advances have facilitated the generation of three-dimensional (3D) neuronal systems and brain assembloids that more closely align with human development and physiology. Indeed, 3D cerebral organoid studies of key neurodevelopmental stages of the human cortex, midbrain, forebrain, hippocampus, striatum and cerebellum (Jo et al., 2016; Birey et al., 2017; Lancaster et al., 2017; Miura et al., 2020; Fear et al., 2022; Kim et al., 2023) have highlighted key differences with animal systems. Long-term maturation of neuronal organoids has allowed better recapitulation of the latter stages of fetal brain development (Giandomenico et al., 2021). Generation of patient-derived neuronal organoids has thus provided new-humanized models of disease (Lancaster et al., 2013; Bershteyn et al., 2017; Iefremova et al., 2017). which can be a unique platform for testing novel therapeutic approaches in a “first-in-human” context, providing another preclinical model for evaluating drug efficacy (Lancaster et al., 2013; Galet et al., 2020). The evaluation of saRNAs in patient-derived organoids could therefore potentially yield important proof-of-concept data with regard to drug delivery, efficacy and off-target toxicity. Moreover, the next-generation of complex organoids include the brain blood barrier, which could also inform on systemic delivery of saRNAs (Bergmann et al., 2018; Ahn et al., 2021).

## Conclusion and future perspectives

Several studies have shown preclinical feasibility of saRNA-based therapies for the treatment of different disorders. Indeed, saRNAs have been shown to be effective in reversing disease-related phenotypes in cell lines and animal models, now leading to the first clinical trial (Voutila et al., 2017; Reebye et al., 2018; Zhou et al., 2019). Technological advances have allowed the development of novel delivery systems for specific targeting of cells *in vivo,* aiming to avoid off-targets effects. Further development and optimization of saRNA for the treatment of several diseases are ongoing (Zhou et al., 2019; Sarker et al., 2020).

In this context, HKMT-NDDs represent a promising group of diseases that could benefit from this strategy, though much work needs to be done preclinically to prove safety and efficacy. Another major point to consider is the therapeutic window for saRNA administration. We still lack knowledge on the underlying cellular and molecular mechanisms underpinning HKMT-related NDDs, and very little is known about the specific developmental stages that are most affected in disease. It is likely that some of these diseases have prenatal onset and so fetal therapy strategies may need to be considered in the future. Nevertheless, as is the case for many early onset genetic disorders (Pearson et al., 2021; Fumagalli et al., 2022; Masson et al., 2022; Strauss et al., 2022), such therapies have the potential to significantly modify disease at different stages of the disease course, which can significantly improve patient lifespan and quality of life.

In summary, saRNAs are an important and relevant therapeutic strategy to explore for HKMT-NDD. The recent clinical trial with MTL-CEBPA for treatment of HKMT-related cancer brings promise and will no doubt provide more knowledge to improve the development of future saRNA-based therapies for NDDs. If the challenges of accurate and targeted brain delivery, repeated dosing, adequate efficacy, optimum therapeutic window, safety, and off-target toxicity can be overcome, saRNA therapies could potentially be applicable to a broad range of monogenic disorders of genetic haploinsufficiency.
